# Influence of the gut microbiota on endometriosis: Potential role of chenodeoxycholic acid and its derivatives

**DOI:** 10.3389/fphar.2022.954684

**Published:** 2022-08-22

**Authors:** Yangshuo Li, Kaili Wang, Jie Ding, Shuai Sun, Zhexin Ni, Chaoqin Yu

**Affiliations:** ^1^ Department of Traditional Chinese Gynecology, The First Affiliated Hospital of Naval Medical University, Shanghai, China; ^2^ Department of Pharmaceutical Sciences, Beijing Institute of Radiation Medicine, Beijing, China

**Keywords:** chenodeoxycholic acid, gut microbiota, endometriosis, inflammation, bile acids

## Abstract

The gut microbiota (GM) has received extensive attention in recent years, and its key role in the establishment and maintenance of health and in the development of diseases has been confirmed. A strong correlation between the GM and the progression of endometriosis (EMS) has been observed in emerging research. Alterations in the composition and function of the GM have been described in many studies on EMS. In contrast, the GM in the environment of EMS, especially the GM metabolites, such as bile acids and short-chain fatty acids that are related to the pathogenesis of EMS, can promote disease progression. Chenodeoxycholic acid (CDCA), as one of the primary bile acids produced in the liver, is metabolized by various enzymes derived from the GM and is critically important in maintaining intestinal homeostasis and regulating lipid and carbohydrate metabolism and innate immunity. Given that the complexity of CDCA as a signalling molecule and the interaction between the GM and EMS have not been clarified, the role of the CDCA and GM in EMS should be understood from a novel perspective. However, few articles on the relationship between CDCA and EMS have been reviewed. Therefore, we review the available and possible potential links between CDCA, the GM and EMS and put forward the hypothesis that CDCA and its derivative obeticholic acid can improve the symptoms of EMS through the GM.

## Introduction

Endometriosis (EMS) is an estrogen-dependent disease characterized by the planting and growth of endometrial stromal cells and gland cells outside the uterine cavity (Zondervan et al., 2020). EMS can be traditionally divided into three types, i.e., peritoneal, ovarian EMS and deep invasive EMS. Rare EMS cases of distal organs, such as thoracic EMS and cerebellar EMS, have been reported in case reports (Meggyesy et al., 2020; Chen and Li, 2021). However, the pathophysiology of EMS is still unclear. The most widely considered and accepted mechanism is the retrograde menstrual mechanism. In addition, the pathogenesis may involve factors such as coelomic metaplasia, immune alterations, stem cell theory, genetics and Mullerian duct residue (Vercellini et al., 2014; Bulun et al., 2019). Women with EMS usually suffer from chronic pain and infertility in the clinic. Given that EMS tends to be confused with digestive diseases, the diagnosis of EMS is generally not timely, thereby seriously affecting the quality of life of patients and causing huge economic losses (Surrey et al., 2020). Therefore, further elucidation of the pathological mechanism of EMS may lay the foundation for the development of new diagnostic and therapeutic drugs.

The gut microbiota (GM) exists in the human gastrointestinal tract and plays a key role in the regulation of human health and disease, and its role is no less than that of other organs in the human body (O'Hara and Shanahan, 2006). The GM is rich in species diversity and abundance, and its number is equivalent to ten times the total number of human cells. *Firmicutes* and *Bacteroidetes* are the main components of the GM and account for more than 90% of the GM composition, and *Firmicutes*/*Bacteroidetes* (F/B) is also an important regulatory factor for maintaining host homeostasis (Turnbaugh et al., 2006). F/B is usually elevated when the GM is disturbed. When host homeostasis is unregulated, the internal environment changes, leading to alterations in the structure of the GM, which in turn influences the host. Therefore, the internal environment and the GM jointly regulate the host’s homeostasis (Rooks and Garrett, 2016). The connections between the GM and the host are not limited to the intestines and involve many organs and diseases throughout the host, such as cardiovascular, nervous, respiratory, liver, autoimmunity, and other diseases (Sharon et al., 2016; Budden et al., 2017; Tang et al., 2017; Clemente et al., 2018; Ma et al., 2018).

Chenodeoxycholic acid (CDCA) is one of the main primary bile acids (BAs) in human and animal bile and is generated from the conversion of cholesterol in liver cells. Initially, CDCA was recognized as a lipid solvent and plays an indispensable role in dissolving cholesterol and maintaining enterohepatic circulation (Chenodeoxycholic acid in the dissolution, 1976). CDCA, as a signalling molecule, can also play an important role in a variety of physiological functions (Song et al., 2019). The signal transduction effect of CDCA plays a considerable role in metabolic diseases and in some inflammation-related diseases and cancers (Thomas et al., 2008; Jia et al., 2018).

Our previous study found that the level of CDCA in the intestine of EMS mice is increased (Ni et al., 1989a) and that CDCA can improve the abundance of the GM of EMS mice, which showed a high degree of similarity with normal mice (not published). However, few articles discussing the complex mechanism of CDCA in EMS in detail have been written. This article reviews the relationship between EMS and GM imbalance, the regulation of CDCA on the GM and inflammatory environment, the possible mechanism by which CDCA alleviates the symptoms of patients with EMS by improving the GM and reducing inflammation, and the potential of CDCA and its derivative obeticholic acid (OCA) in the treatment of patients with EMS.

## Relationship between EMS and GM imbalance

Studies have shown that EMS changes the structure of the GM, causes an imbalance in the F/B ratio, and reduces the α and β diversity (Ni et al., 1989a; Ni et al., 1989b), which are measures of the diversity of the microbiota within a single individual and an indicator to characterize the similarities in microbial composition between individuals, respectively (Table 1). The imbalance in the GM leads to inflammation that damages the health of the intestinal epithelium and affects the health of the whole body through the downstream effects of metabolism. Decreased abundance of Lachnospiraceae and *Ruminococcus* in EMS has been reported in several studies (Ni et al., 1989a; Huang et al., 2021), and the two microbiotas modulate host physiology and immunity by production of short-chain fatty acids (SCFAs) (Zhang et al., 2021). SCFA, a GM metabolite, has been further demonstrated to have an ameliorative effect on EMS by inhibiting human endometriosis cell survival and lesion growth through G protein-coupled receptors, histone deacetylase and GTPase activating protein RAP1GAP (Chadchan et al., 2021). In addition, Lachnospiraceae can also promote the conversion of primary BAs to secondary BAs (Sorbara et al., 2020). Ruminococcaceae family was decreased in some reports (Yuan et al., 2018; Cao et al., 2020). *Ruminococcus gnavus* has been shown to produce inflammatory polysaccharides, which are closely associated with Crohn’s disease and to modulate the differentiation of T helper cells that express IL-17A (Th17 cells) through the production of specific secondary BA derivatives (Henke et al., 2019; Paik et al., 2022). Studies have reported that IL-17A plays an important role in EMS progression (Shi et al., 2022). *Lactobacillus* has long been used as a probiotic supplement to maintain health as well as prevent and treat diseases, but its proportion in GM elevated in EMS models (Ni et al., 1989b; Cao et al., 2020), which may be due to suppression of the differentiation of Th17 cells by *Lactobacillus* (Wilck et al., 2017)*.* Furthermore, we found that the proportion of bifidobacteriaceae was elevated in some reports (Ni et al., 1989b; Yuan et al., 2018; Shan et al., 2021), and a high proportion of bifidobacteriaceae promoted the differentiation of Th17 cells (Ang et al., 2020). Furthermore, feces from mice with EMS were transplanted into antibiotic gavaged EMS mice which exhibited more severe EMS symptoms, such as an increased number of endometriotic foci (Chadchan et al., 2021). Taken together, the progression of EMS is further promoted by the disturbed GM in EMS patients or mice, the mechanisms of which are multifaceted and probably involve metabolites and Th17 cell differentiation.

**TABLE 1:** The GM in EMS studies.

Subjects	Phylum	Class	Order	Family	Genus	Species	F/B ratio	References
Mouse	*Proteobacteria* ↑	N/A	N/A	N/A	*Allobaculum*↑	N/A	↓ (*p* > 0.05)	Ni et al. (1989a)
	*Verrucomicrobia* ↑				*Akkermansia*↑			
					*Parasutterella*↑			
					*Rikenella*↑			
					*Lachnospiraceae*_NK4A136_group↓			
					*Lactobacillus*↓			
					*Bacteroides*↓			
Mouse	*Actinobacteriota*↑	N/A	N/A	N/A	*Lactobacillus*↑	N/A	↑	Ni et al. (1989b)
	*Patescibacteria*↑				*Clostridium*_sensu_stricto_1↑			
	*Deferribacterota*↓				*Bifidobacterium*↑			
	*Campilobacterota*↓				*Candidatus_Saccharimons*↑			
	*Desulfobacterota*↓				*Bacteroides*↓			
					*Dubosiella*↓			
					*Muribaculum*↓			
Human	N/A	N/A	*Clostridiales*↓	*Lachnospiraceae*↓	*Ruminococcus*↓	*Eggerthella lenta* ↑	N/A	Huang et al. (2021)
						*Eubacterium dolichum*↑		
Mouse	N/A	Bacilli↑	N/A	*Lactobacillaceae*↑	*Lactobacillus*↑	N/A	↑	Cao et al. (2020)
		Clostridia↓		*Ruminococcaceae*↑				
		*Bacteroidia*↓		*Peptostreptococcaceae*↓				
				*Ruminococcaceae*↑				
Mouse	*Actinobacteria*↑	*Actinobacteria*↑	*Bifidobacteriales*↑	*Bifidobacteriaceae*↑	*Ruminococcaceae*-UGG-014 ↑	N/A	↑	Yuan et al. (2018)
		*Betaproteobacteria*↑	*Burkholderiales*↑	*Alcaligenceae*↑	*Bifidobacterium*↑			
		*Bacteroidia*↓	*Bacteroidales*↓	*Bacteroidales*↓	*Parasutterella*↑			
Human	*Actinobacteria*↑	N/A	N/A	N/A	*Bifidobacterium*↑	N/A	↑	Shan et al. (2021)
	*Cyanobacteria*↑				*Blautia*↑			
	*Saccharibacteria*↑				*Dorea*↑			
	*Fusobacteria*↑				*Streptococcus*↑			
	*Acidobacteria*↑				[*Eubacterium*] *hallii_group*↑			
					*Lachnospira*↓			
					[*Eubac-terium*] *eligens_group*↓			
Human	N/A	N/A	N/A	N/A	*Sneathia*↓	N/A	N/A	Ata et al. (2019)
					*Barnesella*↓			
					*Gardnerella*↓			
Mouse	N/A	N/A	N/A	N/A	*Bacteroides*↑	N/A	↓	Chadchan et al. (2019)
Human	N/A	N/A	N/A	N/A	*Clostridium*↑	N/A	↑	Le et al. (2021)
					*Bacteroides*↓			
					*Prevotella*↓			
Human	N/A	N/A	*Bacteroides*↑	N/A	*Turicibacter*↓	N/A	N/A	Svensson et al. (2021)
			*Parabacteroides*↑		*Lachnospira*↑			
			*Oscillospira*↑		*Oscillospira*↑			
			*Coprococccus*↑					
			*Paraprevotella*↓					

Note: N/A, not applicable. GM, gut microbiota; EMS, endometriosis.

**FIGURE 1:**
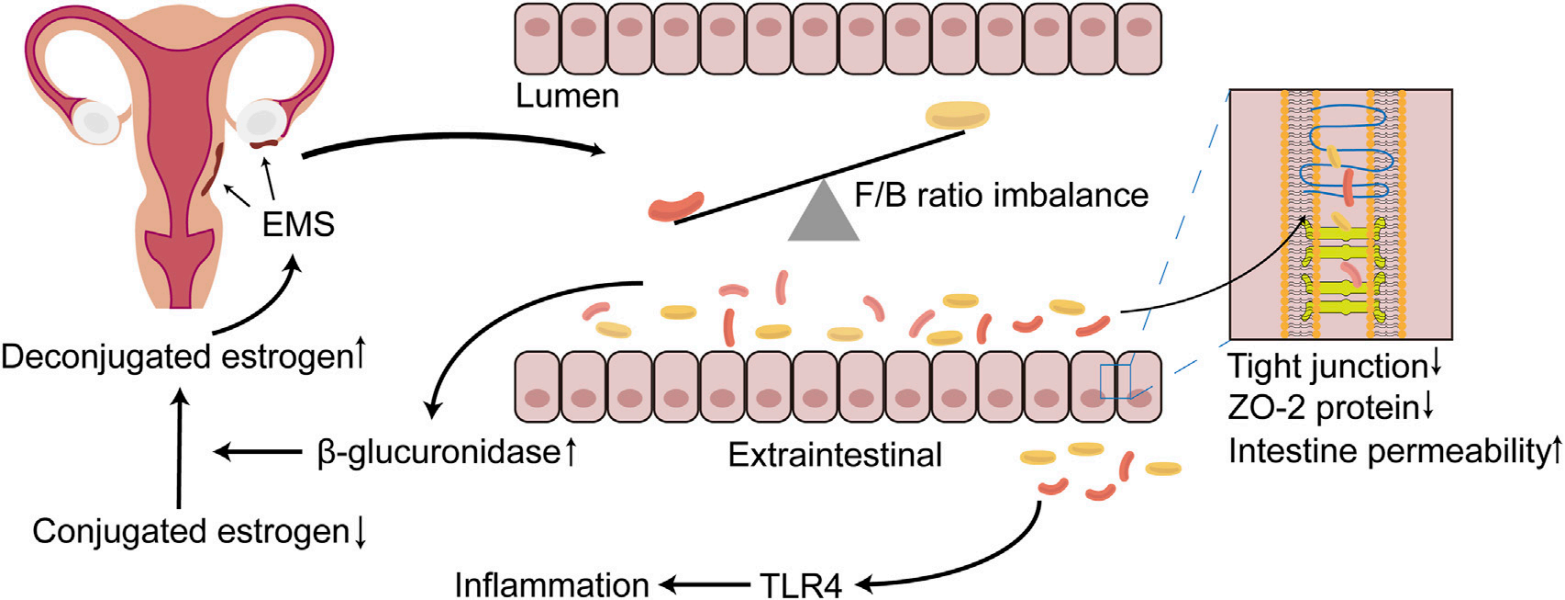
The relationship between EMS and GM imbalance. During the progression of EMS, which can occur the imbalance of GM, the structure and the diversity of GM are altered, with the most dominant Firmicutes/Bacteroidetes (F/B) ratio being dysregulated. The disorder of GM results in the destruction of tight junction, the reduction of the expression of ZO-2 protein and further the increasing of the permeability of the intestine. Bacteria penetrate outside the intestinal lumen, activating TLR4, and lead to inflammatory responses, which may create a suitable environment for EMS onset and progression. In addition, β-glucuronidase may be increased in the intestine due to the imbalance of the GM, rendering more unconjugated estrogen that is transported to the EMS lesion location *via* blood vessels, binding to the estrogen receptor, further promoting the progression of EMS. EMS, endometriosis; GM, gut microbiota; F/B, Firmicutes/Bacteroidetes; TLR4, toll-like receptor 4.

The GM promotes the progression of EMS, possibly because the GM participates in the metabolism of estrogen. β-Glucuronidase is secreted by the GM and can metabolize BA-secreted conjugated estrogen and phytestrogens to the deconjugated form (Baker et al., 2017; Ervin et al., 2019). These deconjugated and unbound forms of estrogen can enter the blood and bind to downstream estrogen receptors to regulate female reproductive development (Baker et al., 2017). The abnormal structure of the GM in EMS may promote the excessive secretion of β-glucuronidase, thereby allowing increased effective estrogen to enter the blood and bind to the estrogen receptor in the ectopic endometrial tissue. This phenomenon inhibits the antagonism of progesterone and promotes the proliferation of focal cells to further aggravate the inflammatory response (Figure 1) (Marquardt et al., 2019). Interestingly, estrogen and its derivatives have been shown to reduce the production of lipopolysaccharide (LPS) by the GM and weaken the permeability of the intestine, thereby maintaining the integrity of the intestinal epithelial barrier (Homma et al., 2005). This finding seems to contradict the ability of estrogen to promote EMS cell proliferation. One explanation is that the increased tolerance of EMS to progesterone increases local estrogen levels and promotes the proliferation of ectopic endometrial lesions (Marquardt et al., 2019).

The theory of menstrual retrograde has received significant attention and is widely recognized in the aetiology of EMS. Menstrual retrograde occurs in 90% of women, but only 10% of women develop EMS, which is caused by several factors. The immune system has been a hotspot that has attracted much attention. Immune dysfunction has been confirmed to be a key factor that promotes the growth of ectopic lesions after the retrograde menstruation of endometrial fragments (Symons et al., 2018; Jeljeli et al., 2020; Agostinis et al., 2021). Many iron-containing haemoglobin and reactive oxygen species (ROS) in menstrual fragments retrograde through the fallopian tube, creating an iron overload environment in the peritoneal cavity. This phenomenon promotes the proliferation and polarization of macrophages (Lousse et al., 2009; Woo et al., 2020). Recent studies have shown that the special microenvironment of EMS may promote the polarization tendency of endometrial M2 macrophages towards M1 polarization and that M2 macrophages paradoxically express proinflammatory phenotypes in the initial phase, whereas the proinflammatory phenotype of M1 macrophages is promoted when endometriotic lesions are established (Vallvé-Juanico et al., 2019). The GM has been shown to regulate extraintestinal inflammation (Karmarkar and Rock, 2013). The GM altered by EMS leads to the destruction of the tight junctions of the intestine and decreases the expression of ZO-2 protein, which further increases the permeability of the intestine (Ni et al., 1989b), resulting in the translocation and infiltration of a large number of Gram-negative bacteria outside the intestinal cavity (Meroni et al., 2019). LPS can activate macrophage Toll-like receptor 4 (TLR4) in innate immunity, producing large amounts of tumour necrosis factor-α (TNF-α) and interleukin (IL)-8 and promoting the formation of an inflammatory environment (Figure 1) (Harada et al., 2001). TNF-α and IL-8 play an essential role in endometrial tissue adhesion and the induction of angiogenesis (Nothnick, 2001). The activation of TLR4 signalling and the inflammatory environment may promote the polarization of M1 macrophages and cause them to express proinflammatory phenotypes (Wanderley et al., 2018). The ability of polarized M1 macrophages to phagocytose haemoglobin and ectopic endometrial tissue in the peritoneal cavity is inhibited, but the promotion of the growth of ectopic endometrial tissue is unrestricted. In addition, excess divalent iron ions in the peritoneal cavity enter ectopic endometrial tissue through divalent metal transporter-1, which promotes an increase in intracellular ROS and acts on NF-κB to promote the proliferation of endometrial stromal cells and inhibit apoptosis (Azuma et al., 1989). Iron may contribute to the migration abilities of human endometriotic cells *via* matrix metalloproteinase expression through the ROS–NF-κB pathway (Woo et al., 2020). These studies showed that the imbalance in the GM promotes the progression of EMS in such ways.

In addition, studies have found that dietary differences through the GM metabolism may also be a contributing factor to EMS. EMS patients consume more red meat, coffee and trans fat but less vegetables and omega-3 polyunsaturated fatty acids (PUFAs) (Parazzini et al., 2013). PUFAs have been confirmed to have anti-inflammatory effects in EMS mouse models (Attaman et al., 1989) and can effectively relieve pain in young women with EMS (Nodler et al., 2020). It can be inferred that the GM with EMS affects part of the host’s dietary habits and metabolizes the diet ingested by the host into products that may further promote the development of EMS. It has been reported that the content of short-chain fatty acids produced by intestinal microbial metabolism in the feces of EMS model mice is less than that of normal mice, but it can effectively prevent the progression of EMS (Chadchan et al., 2021). Therefore, EMS patients could suppress disease progression by adjusting their diet, such as eating more fish oil possessing anti-inflammatory effects and consuming beneficial bacteria that can metabolize and produce short-chain fatty acids.

In short, a close relationship between the imbalance in the GM and the pathogenesis and progression of EMS has been identified, and various complex mechanisms of the relationship urgently need to be confirmed in future studies.

## CDCA regulates GM structure and inflammation

**FIGURE 2:**
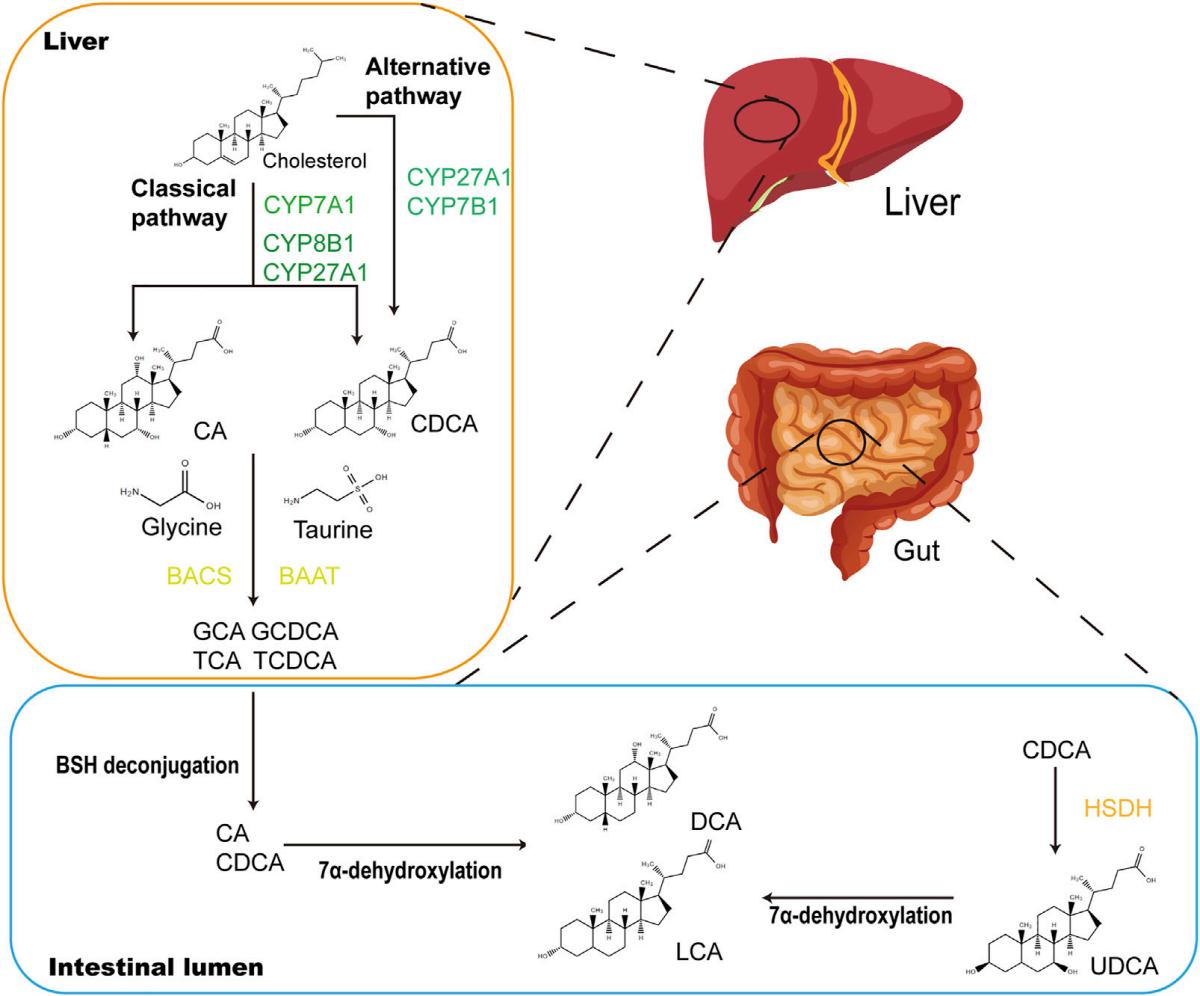
CDCA biosynthetic and metabolic pathways. In the liver, CDCA are synthesized from cholesterol *via* oxidation catalyzed by CYPs, which occurs in the classical and alternative pathways. In the classical pathway, CYP7A1 converts cholesterol to 7*α*-hydroxycholesterol, which is then transformed into CA and CDCA through the subsequent actions by CYP8B1 and CYP27A1. The alternative pathway is initiated by CYP27A1 and produces CDCA by CYP7B1. Primary BAs CA and CDCA are amidated with glycine or taurine to form conjugated bile acids (GCA, GCDCA, TCA and TCDCA) under the actions of BACS and BAAT. When secreted into the gut after meals, conjugated bile acids are transformed into secondary BAs (LCA and DCA) *via* biotransformation of GM. Major microbial biotransformation reactions include deconjugation mediated by BSH, 7α-dehydroxylation and oxidation and epimerization mediated by HSDH. CYP, cytochrome P450; CA, cholic acid; GCA, glycocholic acid; GCDCA, glycochenodeoxycholic acid; TCA, taurocholic acid; TCDCA, taurochenodeoxycholic acid; BACS, bile acyl-CoA synthetase; BAAT, bile acid-CoA:amino acid N-acyltransferase; LCA, lithocholic acid; DCA, deoxycholic acid; BSH, bile salt hydrolase.

CDCA is one of the main primary BAs in humans and animals. Primary BAs consist of CDCA, cholic acid and conjugated BA (CBA) (Figure 2). CDCA is conjugated with taurine or glycine to form CBA in the liver. After each meal, BAs are secreted into the upper small intestine. In the intestinal lumen, GM-mediated bile salt hydrolase (BSH) deconjugation converts CBA into unconjugated BAs. The distribution of BSH in the GM is only detected in members of the phylum *Bacteroidetes*, including *Clostridium*, *Enterococcus*, *Bifidobacterium*, and *Lactobacillus* (Cai et al., 2022). 7α-Dehydroxylation mediated by *Clostridium* and *Eubacterium* converts primary BAs into secondary BAs by removing the 7a/b-hydroxy group (Jia et al., 2018). Furthermore, CBA activates pancreatic lipase and forms mixed micelles with monoglycerides, cholesterol, some free fatty acids and fat-soluble vitamins to promote their absorption by intestinal cells. Unconjugated BAs and some glycine-CBAs are reabsorbed in the small intestine through passive diffusion (Dawson and Karpen, 2015). This process is called “enterohepatic circulation of BAs” and has physiological significance because it regulates the synthesis of BAs through feedback inhibition to absorb and transport cholesterol, fat and nutrients to the liver and distribute them to other organs (Hofmann, 2009).

**FIGURE 3:**
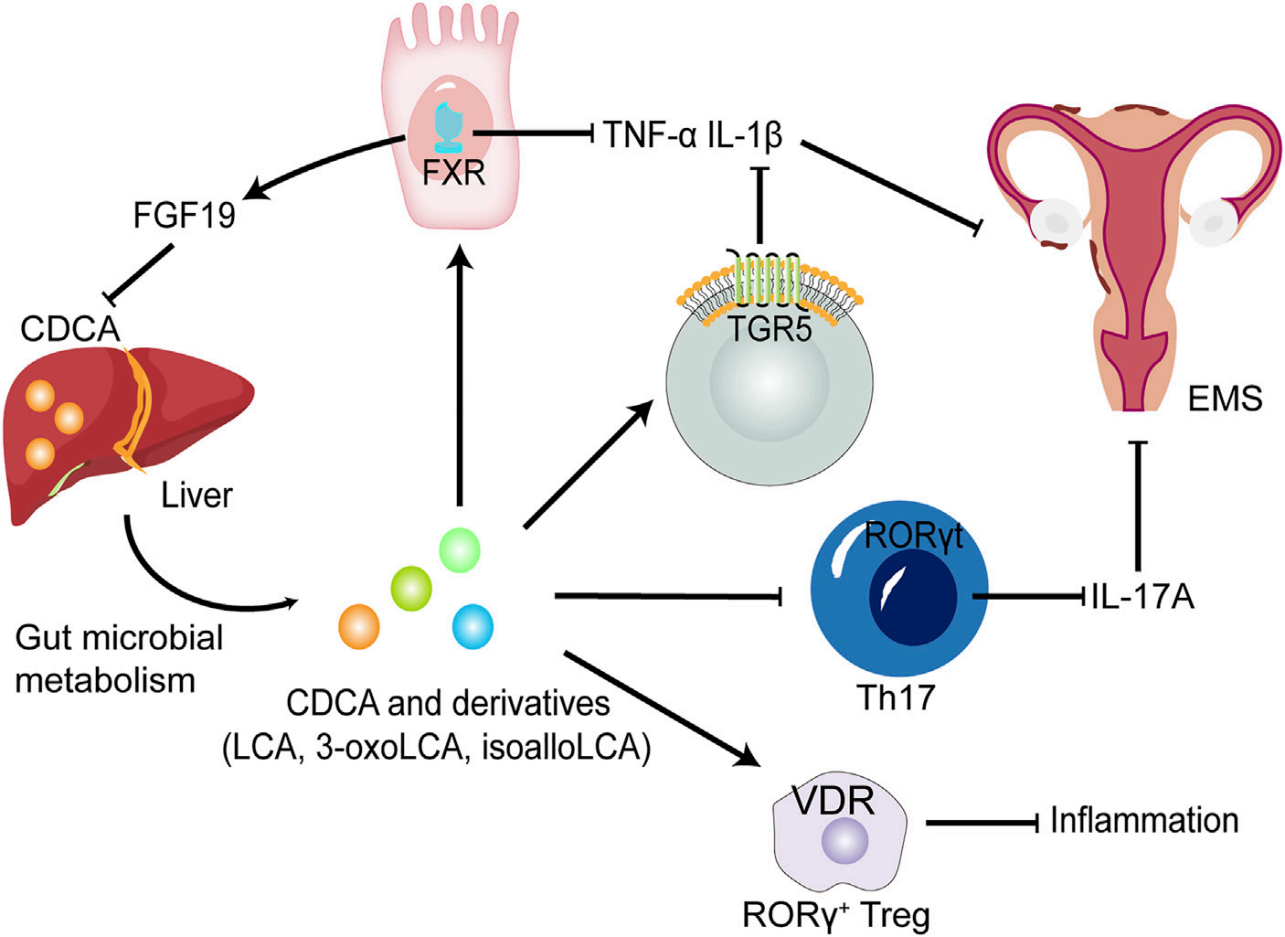
The potential roles of CDCA and derivatives in EMS. CDCA produced from the liver is converted into LCA, 3-oxoLCA and isoLCA after being metabolized by GM. FXR and TGR5 activated by CDCA and derivatives suppress proinflammatory cytokine expression, such as IL-1β and TNF-α in dendritic cells and macrophages. CDCA promote the release of FGF-19 by activating FXR, thereby inhibiting the production of CDCA, further forming a negative feedback regulation mechanism in normal environment. 3-oxoLCA and isoLCA inhibit the differentiation of Th17 cells expressing IL-17A upon direct binding to RORγt. Decreased levels of proinflammatory cytokines could improve EMS. Furthermore, CDCA, LCA and 3-oxoLCA modulate RORγ Tregs through the BA receptor VDR to suppress inflammation. FGF-19, fibroblast growth factor 19; RORγt, retinoic acid-related orphan receptor γt; Treg cells, regulatory T cells; VDR, vitamin D receptor.

As a signalling molecule, CDCA can activate the two main BA-sensitive receptors, i.e., BA sensor farnesoid X receptor (FXR) and G protein-coupled BA receptor GPBAR1 (TGR5), and exert endocrine functions (Figure 3). TGR5 is a membrane receptor that is predicted to have 7 transmembrane domains and is a 330-amino-acid-long protein. TGR5 is widely distributed and can be expressed in a variety of organs and cell types, including the gallbladder, intestinal tract, immune cells, fat cells, muscles and nervous systems (Maruyama et al., 2002; Kawamata et al., 2003). Among the natural TGR5 agonists, CDCA is the agonist with the most affinity for TGR5 except lithocholic acid (LCA) and deoxycholic acid. The negative regulation of inflammation by TGR5 is found in macrophages in liver cells (Kupffer cells). BAs inhibit LPS-induced cytokine production by activating TGR5 in macrophages, thereby improving the inflammatory environment (Keitel et al., 2008). Therefore, the activation of the TGR5 signalling pathway can have a beneficial effect on a variety of inflammatory diseases. In intestinal inflammatory diseases, macrophages, which are essential for intestinal immune homeostasis, are the phagocytic cells that interact with microorganisms and microbial products and have a dual role in protecting the host from pathogens and regulating the mucosal response to symbiotic bacteria (Smythies et al., 2005). Inflammatory bowel disease (IBD) is caused by a disproportionate immune response to antigens in the intestinal cavity, leading to chronic inflammation of the intestine (Zhang and Li, 2014). The deletion of TGR5 easily aggravates intestinal inflammation in a mouse model of colitis, whereas the activation of TGR5 reduces the local expression of inflammatory cytokines (Cipriani et al., 2011). In contrast, the activation of TGR5 *in vivo* can promote the polarization of intestinal mucosa-related immune macrophages from M1 to M2, thereby reducing inflammation in mice (Biagioli et al., 2017). In addition, TGR5 agonists can induce monocytes to differentiate into IL-12 and TNF-α hyposecretory dendritic cells through the TGR5-cAMP signalling pathway (Ichikawa et al., 2012). TGR5, as an indispensable receptor in macrophages, can be activated by BAs, which can transform macrophages into an M2-like phenotype and cause macrophages to exert immunosuppressive behaviour (McMahan et al., 2013). LCA, as the most potent activator of TGR5, is metabolized by CDCA through the GM. Thus, the GM may regulate the activation of TGR5 to a certain extent. The structure of the GM changes in the EMS environment, which may result in the inability of CDCA to be effectively converted into LCA through the GM and in the downregulation of TGR5 signal efficiency. Macrophages play a key role in the progression of EMS, and M2 macrophages activated by TGR5 signalling can reduce inflammation and enhance immunosuppression. Excitingly, a study revealed that TGR5 was expressed in human endometriotic stromal cells, and INT-777, a potent agonist of TGR5, could significantly reduce the production of proinflammatory cytokines and adhesion molecules by inhibiting TNF-α, suggesting that TGR5 activators have beneficial effects against inflammation and ROS in cytokine-induced activation of endometriotic stromal cells (Lyu et al., 2019).

FXR was the first BA nuclear receptor discovered, and CDCA has the highest affinity among its natural ligands (Ridlon et al., 2014). After activation, FXR can inhibit the expression of the apical sodium-dependent bile acid transporter (ASBT) and increase the expression of two transporters, i.e., organic solute transporter α (OSTα) and β (OSTβ), to reduce the uptake of BA, promote the secretion of BA in basal cells and reduce the concentration of BA in basal cells (Ding et al., 2015). Moreover, FXR is involved in intestinal immune regulation and intestinal mucosal barrier function, reduces inflammation and maintains the integrity of the intestinal epithelial barrier by regulating the degree of inflammation, maintaining the integrity and function of the intestinal wall barrier, and preventing bacterial translocation in the intestine (Gadaleta et al., 2010; Gadaleta et al., 2011a; Sorribas et al., 2019). Vavassori et al. noticed that the expression of proinflammatory cytokine mRNA in the colon of FXR-deficient mice was increased (Vavassori et al., 2009). Raybould et al. found that the activation of FXR by INT-747 can prevent DSS- and TNBS-induced intestinal inflammation, improve colitis symptoms, inhibit epithelial permeability, and reduce goblet cell loss (Raybould, 2012). Inagaki et al. proved that intestinal FXR plays a key role in limiting the overgrowth of bacteria and found that the bacteria in the intestine of mice lacking FXR overgrow, thereby increasing the permeability of the intestine (Inagaki et al., 2006). Studies have found that the activation of the NF-κB pathway leads to increased expression of proinflammatory cytokines, thereby reducing the transcription of FXR target genes and further exacerbating inflammation (Gadaleta et al., 2011b). Interestingly, unconjugated BAs, which can cause damage to liver cells at high concentrations, can regulate intestinal barrier function by activating FXR to regulate innate immunity. Therefore, an appropriate concentration of CDCA to regulate the GM is important.

Receptors that respond to CDCA and its derivatives also include vitamin D receptor (VDR) and retinoic acid-related orphan receptor γt (RORγt) (Figure 3). The antiproliferative and anti-inflammatory effects of VDR in EMS are gradually being revealed. VDR agonists reduce IL-1β- and TNFα-induced inflammatory responses and decrease metalloproteinase (MMP)-2 and MMP-9 mRNA levels in ectopic endometrial tissue (Delbandi et al., 1989). In addition, *in vitro* experiments also revealed that VDR activation control the inflammatory response mediated by TLR4 and TLR2 in endometrial stroma (Ghanavatinejad et al., 2021). RORγt is a key transcription factor regulating Th17 cell differentiation (Paik et al., 2022). It has been reported that Th17 cells and IL-17A secreted by Th17 cells play an important role in the progress of EMS (Shi et al., 2022). Furthermore, following direct binding of 3-oxoLCA to RORγt, a reduced differentiation of Th17 cells in the mouse intestinal lamina propria was observed (Paik et al., 2022), suggesting that CDCA derivative-regulated RORγt transcription factors could serve as a potential therapeutic strategy for EMS.

CDCA, can maintain its own metabolic function through the enterohepatic circulation pathway and can directly interact with the microbiota in the intestine as an antibacterial agent. As a signalling molecule, CDCA activates the nuclear receptor and membrane receptor, thereby improving the structure of the GM and intestinal barrier function, inhibiting the secretion of inflammatory cytokines, and reducing the inflammatory environment.

## The relationship between CDCA, GM and EMS

The GM of patients with EMS has changed, and this change is caused by many factors, as mentioned above. Compared with those of control patients, the α and β diversity values of the GM of patients decreased to varying degrees. The abundance values of various bacterial groups measured in different experiments also vary, probably due to discrepancies in sequencing methods and experimental details (Ni et al., 1989a; Ni et al., 1989b; Ata et al., 2019; Chadchan et al., 2019; Le et al., 2021; Svensson et al., 2021). Nevertheless, these alterations in the GM can be considered the body’s response to EMS. The anabolism of BAs is a process involving self-feedback regulation in which FXR in the intestine and liver plays an essential role. The activation of intestinal FXR can upregulate mouse fibroblast growth factor (FGF)-15 and human FGF-19 to activate liver FGF receptor 4 (FGFR4) and inhibit the activity of liver cholesterol 7α-hydroxylase, a rate-limiting enzyme for the synthesis of CDCA and BAs (Gadaleta et al., 2015; Chambers et al., 2019). CDCA can activate intestinal FXR, which inhibits the continuous synthesis of CDCA through this negative feedback mechanism. An imbalance in the F/B ratio is widely regarded as a characteristic of GM dysbiosis (Turnbaugh et al., 2006). The GM has been found to display an imbalance in the F/B ratio in EMS (Ni et al., 1989a; Ni et al., 1989b; Ata et al., 2019; Chadchan et al., 2019), which has caused a series of metabolic disorders in the body. The imbalance in the F/B ratio may prevent CDCA from being metabolized into secondary BAs by related enzymes derived from the GM, causing CDCA to accumulate in the intestine. The abnormal F/B ratio may increase the expression of tauro-β-muricholic acid, a bile salt in the intestine of mice that is an effective antagonist of FXR. Tauro-β-muricholic acid inhibits FXR activation, enhances the synthesis of CDCA in the liver and affects enterohepatic circulation. These phenomena result in an increase in the level of CDCA (Sayin et al., 2013). In addition, the probiotic *Lactobacillus rhamnosus* GG (LGG) is reported to inhibit the synthesis of BAs and to promote the excretion of BAs through the intestinal FXR–FGF-15 signalling pathway (Liu et al., 2020), indicating that probiotics can reverse the imbalance in the GM and BA metabolism to a certain extent. A series of experiments have discussed the close relationship between CDCA metabolism and the GM. Although the experiments are not without limitations, they also provide a feasible explanation for how EMS indirectly affects the metabolic level of CDCA after changing the structure and diversity of the GM. Overall, CDCA has a two-way effect in the intestinal tract. The positive effect of CDCA is that it acts as a signalling molecule to activate related receptors to improve inflammation and reduce BA production, and its passive effect is that it decreases secondary BAs and an excess of primary BAs in the imbalanced GM in individuals with IBD (Sokol et al., 2009), which may lead to a decrease in FXR activity and the downregulation of OSTα and OSTβ expression levels. This phenomenon causes an accumulation of BAs in liver and intestinal mucosal cells, further aggravating the inflammatory phenotype (Gadaleta et al., 2011b; Ding et al., 2015). CDCA and the GM influence each other and jointly produce a gastrointestinal disease phenotype when disrupted. Therefore, in the treatment of gastrointestinal inflammatory diseases, alternatives to CDCA should be sought to reduce its side effects.

In addition, studies have reported that ectopic endometrial cell hyperproliferation in the pelvic cavity of EMS patients as well as triggering symptoms of pelvic pain result from elevated estrogen levels (Marquardt et al., 2019). The bidirectional interaction of high levels of estrogen in the EMS with the GM has been discussed above. Bile acid metabolism has recently been shown to influence estrogen levels, which are simultaneously regulated by bile acid receptor activation and the GM. Cholestatic bile acid levels induced FXR receptor activation and downregulated expression of the hepatic sulfotransferase SULT1E1, increasing levels of estrogen in the serum of mice (Liu et al., 2018). However, another study showed that appropriate concentrations of CDCA and DCA can reduce ESR1 expression in ovarian cancer cells by almost 90% (Jin et al., 2018), indicating that CDCA may reduce proliferation by inhibiting ESR1 expression in ectopic endometrial cells through targeted effects. Furthermore, subcutaneous administration of estrogens to mice induces cholestasis (Dong et al., 2019), which partially explains the abnormal levels of bile acid metabolism in the intestine of EMS mice. However, the mechanisms linking bile acid metabolism and estrogen levels in EMS remain unclear.

Recent studies have shown that IL-17 levels are generally elevated in peritoneal fluid and serum in women with EMS (Shi et al., 2022). IL-17 further leads to proliferation, growth and invasion of endometriotic lesions and promotes immune escape of ectopic lesions and the progression of EMS by inducing M2 macrophage differentiation (Shi et al., 2022). There are several factors that can cause an elevation in IL-17 levels. Specific BA derivatives have been found to act as key players in the control of IL-17-secreting Th17 cells. Two distinct derivatives of LCA, namely, 3-oxoLCA and isoLCA, inhibited the differentiation of Th17 cells by directly binding to the key transcription factor RORγt (Hang et al., 2019; Paik et al., 2022). In addition, the GM from an array of families within the *Actinobacteria* and *Firmicutes* phyla produces 3-oxoLCA, and hydroxysteroid dehydrogenases encoded by *E. lenta* and *R. gnavus* produce 3-oxoLCA and isoLCA (Paik et al., 2022). In summary, as a metabolite of CDCA, LCA is further converted into 3-oxoLCA and isoLCA under the biotransformation of specific GM, and the two derivatives may decrease the level of IL-17 in EMS. On the other hand, isoalloLCA, another LCA derivative, increases regulatory T cell (Treg cell) differentiation *via* producing mitochondrial reactive oxygen species (mitoROS) that promote FOXP3 expression (Hang et al., 2019). Another study reported that CDCA and its derivatives including (LCA and 3-oxoLCA) regulate RORγ^+^ Treg cells through the nuclear receptor VDR (Song et al., 2020). And an imbalance in the Th17/Treg cell ratio leads to ectopic endometrial lesion survival and implantation (Shi et al., 2022). Therefore, specific primary and secondary BAs regulate the Th17/Treg cell ratio, which may influence the progression of EMS.

From the perspective of metabolism, CDCA, as a lipid solubilizer or as a signalling molecule that activates different receptors, can regulate body homeostasis and maintain metabolic balance. CDCA can dissolve cholesterol stones, reduce total cholesterol in bile, and improve glucose homeostasis and lipid and lipoprotein metabolism (Tint et al., 1986; de Aguiar Vallim et al., 2013). In patients and mice with EMS, manifestations, such as a reduction in human adipose stem cells and lipid metabolism disorders in mice, are related to abnormal lipid metabolism (Dutta et al., 2016; Zolbin et al., 2019). Therefore, CDCA may also regulate lipid metabolism to produce PUFAs to exert its anti-inflammatory effect or accelerate fat breakdown to provide the body with the necessary energy to adapt to the inflammatory response.

## Potential therapeutic targeting of OCA in EMS

6-Alpha-ethyl-chenodeoxycholic acid (OCA), which was first discovered while studying FXR receptor ligands, is obtained by adding an ethyl group to the 6-position of the benzene ring of CDCA and has improved physicochemical properties (Pellicciari et al., 2002). Subsequent studies have found that the efficiency of OCA in the activation of FXR is 16–33 times that of the natural ligand CDCA (Pellicciari et al., 2002), and the efficiency in activating TGR5 is close to that of LCA as the natural ligand (Rizzo et al., 2010). In animal experiments, OCA protected against DSS-induced damage in mice, reduced the severity of the disease, and maintained the integrity of the intestinal epithelial barrier (Gadaleta et al., 2011a). At present, OCA was one of the BA receptor modulators approved by the FDA for the clinical treatment of primary biliary cholangitis and effectively reversed or reduced the degree of liver damage in 2016 (Nevens et al., 2016). In a phase III clinical trial of OCA for the treatment of nonalcoholic steatohepatitis (NASH), OCA effectively improved liver fibrosis in patients (Younossi et al., 2019). In recent years, studies have shown that OCA can change the characteristics of the GM of mice and humans and reduce the level of endogenous BAs by activating FXR (Friedman et al., 2018). A high-fat diet can induce damage to the intestinal epithelial barrier and intestinal vascular barrier in mice, leading to the translocation of intestinal bacteria or bacterial products into the blood circulation. OCA can drive the activation of β-catenin in endothelial cells to prevent damage to the intestinal epithelial and intestinal vascular barriers and the progression of NASH (Mouries et al., 2019). In addition, OCA can improve the complications associated with the metabolic disorder caused by GM-related high-fat and high-glucose diets, thereby reversing intestinal barrier dysfunction and restoring serum LPS to normal levels (Wu et al., 2021). Furthermore, the TGR5 agonist INT-777 showed therapeutic implications for EMS by mitigating the inflammatory response in human endometriotic stromal cells (Lyu et al., 2019).

## Conclusion

Intestinal dysbiosis destroys normal immune function and leads to chronic inflammation by changing the spectrum of immune cells and promoting the release of inflammatory cytokines. This chronic inflammatory state creates an ideal environment conducive to increased tissue adhesion and angiogenesis, which may lead to the development of EMS. Moreover, EMS can induce changes in the structure of the GM and possibly increase the abundance of potentially pathogenic species, which may cause a vicious cycle between intestinal dysbiosis and EMS. Abnormal estrogen metabolism and changes in the levels of macrophages and inflammatory cytokines provide a possible explanation for the vicious cycle between EMS and intestinal dysbiosis, but several unknowns related to the GM remain. CDCA can regulate the intestinal immune system and GM through its action as a signalling molecule. In this review, we propose the possibility of CDCA and its derivatives as targeted therapeutic drugs for EMS and theoretically discuss the role of CDCA in EMS *via* the biotransformation of the GM and BA receptors. Several potential approaches that CDCA and its derivatives to treatment of EMS are amelioration of the disordered GM by activating BA receptors and regulation of Th17/Treg cells by CDCA derivatives binding to RORγt and promoting mitoROS production. Furthermore, CDCA can regulate the intestinal inflammatory environment and the GM, which may further improve the activity of β-glucosidase that restores normal levels of estrogen. In addition, effort is still needed in the future to determine the “core” GM that plays a central role in EMS and to clarify the mechanism underlying the relationship between BAs/BA receptor agonists and EMS.
